# Decomposability and mental representation of French verbs

**DOI:** 10.3389/fnhum.2015.00004

**Published:** 2015-01-20

**Authors:** Gustavo L. Estivalet, Fanny E. Meunier

**Affiliations:** ^1^Centre National de la Recherche Scientifique UMR5304, Laboratoire sur le Langage, le Cerveau et la CognitionLyon, France; ^2^Université de Lyon, Université Claude Bernard Lyon 1Lyon, France

**Keywords:** morphology, regularity, decomposition, lexical access, frequency effects, verb inflection

## Abstract

In French, regardless of stem regularity, inflectional verbal suffixes are extremely regular and paradigmatic. Considering the complexity of the French verbal system, we argue that all French verbs are polymorphemic forms that are decomposed during visual recognition independently of their stem regularity. We conducted a behavioral experiment in which we manipulated the surface and cumulative frequencies of verbal inflected forms and asked participants to perform a visual lexical decision task. We tested four types of verbs with respect to their stem variants: a. fully regular (*parler* “to speak,” *[parl-]*); b. phonological change e/E verbs with orthographic markers (*répéter* “to repeat,” *[répét-]* and *[répèt-]*); c. phonological change o/O verbs without orthographic markers (*adorer* “to adore,” *[ador-]* and *[adOr-]*); and d. idiosyncratic (*boire* “to drink,” *[boi-]* and *[buv-]*). For each type of verb, we contrasted four conditions, forms with high and low surface frequencies and forms with high and low cumulative frequencies. Our results showed a significant cumulative frequency effect for the fully regular and idiosyncratic verbs, indicating that different stems within idiosyncratic verbs (such as *[boi-]* and *[buv-]*) have distinct representations in the mental lexicon as different fully regular verbs. For the phonological change verbs, we found a significant cumulative frequency effect only when considering the two forms of the stem together (*[répét-]* and *[répèt-]*), suggesting that they share a single abstract and under specified phonological representation. Our results also revealed a significant surface frequency effect for all types of verbs, which may reflect the recombination of the stem lexical representation with the functional information of the suffixes. Overall, these results indicate that all inflected verbal forms in French are decomposed during visual recognition and that this process could be due to the regularities of the French inflectional verbal suffixes.

## Introduction

The surface frequency effect, which reflects differences in word recognition as a function of form frequency, is one of the most reliable phenomena described in the psycholinguistic field in the last 35 years (Taft and Forster, 1975; Taft, 1979, 2004; Burani et al., 1984; Meunier and Segui, 1999b; Domínguez et al., 2000). Polymorphemic words, in addition to their surface frequency, are characterized by their cumulative frequency (also called lemma frequency), which is defined as the sum of the frequencies of all affixed words that carry that stem (e.g., for the stem *[parl-]*, the sum of the surface frequency of *parlons* “we speak” + the surface frequency of *parlez* “you speak” + the surface frequency of *parlent* “they speak,” etc.). Therefore, word and morpheme frequencies are directly related to the time spent for word recognition, with more frequent words being recognized faster than less frequent ones (Taft and Forster, 1975). The effects of the different frequencies of polymorphemic words are of great interest in the investigation of morphemic representations in the mental lexicon and morphological decomposition during word processing (Colé et al., 1989; Domínguez et al., 2000), especially in languages with rich and paradigmatic morphological systems. The cumulative frequency effect is interpreted as reflecting a decomposition process and shows the influence of the morpheme frequency in retrieval and lexical access (Taft and Forster, 1975; Taft, 2004), whereas the surface frequency effect is interpreted as reflecting either the time spent to retrieve and access a whole word in the mental lexicon (Manelis and Tharp, 1977; Butterworth, 1983) or the morphosyntactic recombination process between stem and affixes (Taft, 1979, 2004).

In this research, we investigated the mental representation of French verb stems, their allomorphy (the alternative forms of a morpheme depending on its phonological and morphological context) and verbal decomposability. Unlike the English verbal system, which is generally divided into two groups (regular and irregular verbs) with few suffixes (i.e., walk[s], walk[ed] and walk[ing]) (Stanners et al., 1979; Aronoff, 1994), the French verbal system has different degrees of stem regularity and a paradigmatic set of suffixes for tenses and agreements. Similarly to other Romance languages (Oltra-Massuet, 1999; Domínguez et al., 2000; Say and Clahsen, 2002; Veríssimo and Clahsen, 2009), French has three groups of verbs (see Table 1). However, in contrast to most Romance languages, the French verbal groups are not explicitly defined in function of the theme vowels. Moreover, French has a particular iambic prosodic system that directly influences the phonetic production of the stems and inflectional suffixes in a predictive way (Aronoff, 2012; Andreassen and Eychenne, 2013). In particular, the pronunciation of the syllables to the right of the stem produces prosodic consequences, which are reflected in phonetic production. Thus, for verbs from the first group that undergo phonological changes, the last vowel of the stem is open pronounced (/E/ and /O/) if the stem is merged with a non-pronounced suffix (e.g., *[-e], [-es], [-ent]* as in *[répèt]e /Re'pEt/* “I/he/she repeat(s)”) but is close pronounced (/e/ and /o/) if the stem is merged with a suffix that has a pronounced vowel (e.g., *[-ons], [-ez], [-ai], [-i], [-er]* as in *[répét]ons /Repe'tõ/* “we repeat”) (Touratier, 1996). A question that remains open is whether different phonological forms of a verb have different lexical representations or whether they share an abstract or underspecified representation.

**Table 1:** **Examples of the three French verbal groups conjugated in the present tense showing the stem regularity and the suffix paradigms**.

***Infinitif***	**1st Group**			**2nd Group**	**3rd Group**		
	***Ramer***	***Céder***	***Moquer***	***Finir***	***Rendre***	***Écrire***	***Devoir***
Je	rame	cède	mOque	finis	rends	écris	dois
Tu	rames	cèdes	mOques	finis	rends	écris	dois
Il	rame	cède	mOque	finit	rend	écrit	doit
Nous	ramons	cédons	moquons	finissons	rendons	écrivons	devons
Vous	ramez	cédez	moquez	finissez	rendez	écrivez	devez
Ils	rament	cèdent	mOquent	finissent	rendent	écrivent	doivent

The first verbal group in French is regular concerning its conjugations and is characterized by the infinitive ending *[-er]*. The second group is also regular and is characterized by the infinitive ending *[-ir]* associated with the realization of the morpheme *[-ss-]* before suffixes beginning with vowels. The third group comprises irregular verbs, including verbs with different infinitive endings (e.g., *[-dre], [-ire], [-oir]*, etc.) and a different number of stems per verb (Kilani-Schoch and Dressler, 2005; Aronoff, 2012). Therefore, the first group has verbs with just one stem, such as *ramer* “to paddle.” The only modification that is observed within a sub-group of stems is a phonological predicted alternation in the stem (these verbs can also be called morpho-phonological verbs), such as the verb *céder* “to cede” (e.g., *[cèd]es /'sEd/* “you cede,” *[céd]ons /se'dõ/* “we cede”) (Halle and Idsardi, 1996; Andreassen and Eychenne, 2013). Stems from the second group are always the same in the full inflectional system (e.g., *[fini]r* “to finish”). Finally, the third group includes verbs with just one stem, such as *rendre* “to render,” verbs with small changes in the stem, such as *écrire* “to write” (e.g., *[écri]t* “he/she writes,” *[écriv]ons* “we write”), and verbs with idiosyncratic stem allomorphs, such as *devoir* “must” (e.g., *[doi]s* “I/you must,” *[dev]ons* “we must,” *[doiv]ent* “they must”) (Touratier, 1996). Unlike stems that carry the lexical meaning, the morphosyntactic inflectional system of tense and agreement suffixes in French is extremely paradigmatic and can be easily detached from the stem to which it is merged (Meunier and Marslen-Wilson, 2004). Thus, all verbal inflected forms in French can be decomposed based on their regular and salient inflectional system of suffixes, and this evident morphosyntactic decomposition may determine the mental representation of verbal stems.

The first objective of the current work was to determine whether the systematic French verbal inflectional system underlies the morphological decomposition of all forms on visual recognition (Rastle and Davis, 2008) or whether inflected verbs can be accessed as whole words. The second objective was to investigate how stems are represented in the mental lexicon in function of their regularity (Bybee, 1995). For this purpose, participants performed a visual lexical decision task on French inflected verbs. We manipulated the surface and cumulative frequencies for four types of stem variants: a. fully regular verbs from the first group (*parler* “to speak,” one form *[parl-]*); b. phonological change e/E verbs from the first group with orthographic markers (*répéter* “to repeat,” two forms *[répét-] /repet-/* and *[répèt-] /repEt/*); c. phonological change o/O verbs from the first group without orthographic markers (*adorer* “to adore,” two forms *[ador-] /ador-/* and */adOr-/*); and d. idiosyncratic verbs from the third group with different stems (*boire* “to drink,” two forms *[boi-]* and *[buv-]*). We tested two different phonological change verbs (i.e., with and without orthographic markers) because the orthographic markers can be a strong hint for phonetic realization (Kilani-Schoch and Dressler, 2005) in visual stimulation, yielding different results (Seidenberg, 1992; Rastle and Davis, 2008).

To explain the word-recognition process, different models have been suggested to account for morphological processing in lexical access. The first type of model proposes an obligatory decomposition process for polymorphemic words upon lexical retrieval and recognition (Halle, 1973; Taft and Forster, 1975; Taft, 1979; Halle and Marantz, 1993; Marantz, 2013) in which the components of polymorphemic words are represented at the form and morphemic levels. The meaning of the whole word form is retrieved when the lexical information of the stem is combined with the morphosyntactic information of the affixes. The second type of model proposes an exclusively associative whole-word lexical access (Manelis and Tharp, 1977; Butterworth, 1983). This type of model includes the connectionist model, with its different variations (Rumelhart and McClelland, 1986; Seidenberg, 1992; Baayen et al., 2011), basically suggesting that morphology emerges from the overlap between meaning, phonology and orthography. The third model type aggregates both decompositional and associative lexical access to propose a dual-route model (Caramazza et al., 1988; Baayen et al., 1997; Clahsen, 1999, 2006; Pinker, 1999).

The dual-route models, such as the Augmented Address Model (AAM) (Burani et al., 1984; Caramazza et al., 1988), the Race Model (RM) (Baayen et al., 1997; Schreuder and Baayen, 1997), and the Words and Rules model (W&R) (Pinker, 1999; Pinker and Ullman, 2002), have been supported by a significant amount of research in different languages in the past few years, with different specifications for each of their versions. However, more specifically for our study, the Minimalist Morphology model (MM) (Wunderlich, 1996) uses the morpheme-based assumption, highlighting the computational route by proposing that regular inflected forms are established by merging constant lexical entries and affixes and that irregular inflected forms are represented by subnodes of lexical entries containing variables (Clahsen, 1999, 2006). Empirical research has been conducted to better understand the general principles of word recognition, including specific morphological parameters that drive the morphological processing and representation in different languages (Beard, 1995). These examinations in verbal inflection have been conducted in English with the now-famous English past tense debate (Stanners et al., 1979; Rumelhart and McClelland, 1986; Marslen-Wilson and Tyler, 1998; Pinker, 1999; McClelland and Patterson, 2002; Pinker and Ullman, 2002; Fruchter et al., 2013), German (Clahsen, 1999), Dutch (Baayen et al., 1997; Schreuder and Baayen, 1997), and Finnish (Leinonen et al., 2008). Romance languages have also been investigated, including Spanish (Domínguez et al., 2000), Catalan (Oltra-Massuet, 1999), Portuguese (Sicuro Corrêa et al., 2004; Veríssimo and Clahsen, 2009), Italian (Burani et al., 1984; Caramazza et al., 1988; Orsolini and Marslen-Wilson, 1997; Say and Clahsen, 2002), and French (Meunier and Marslen-Wilson, 2004; Meunier et al., 2008, 2009).

Altogether, the literature clearly shows that morphological processing has a fundamental role in lexical access, especially in inflected polymorphemic words in which the computational system and the mental lexicon interact for word recognition (Halle, 1973; Colé et al., 1997; Marslen-Wilson and Tyler, 1998; Clahsen, 1999). Concerning verbal form identification, findings in English, Dutch, and German are clear, with multiple sources of evidence in favor of a lexical associative process for irregular words and a rule-based process for regular ones. These findings suggest that regular inflected words are completely combinatorial, whereas irregular inflected words are internally structured and represented in the mental lexicon (Wunderlich, 1996; Baayen et al., 1997; Pinker, 1999). However, based on a facilitatory priming effect for irregular pairs such as *fell—fall* in masked priming, Crepaldi et al. (2010) recently challenged the idea of an exclusively semantic relationship between the irregularly inflected forms and their base forms (see also Forster et al., 1987). These authors proposed a shared representation that underlines both forms at the lemma level where inflected words share their representation irrespective of orthographic regularity (McCormick et al., 2008; Crepaldi et al., 2010). The results observed within Romance languages with a richer verbal morphology are somewhat more puzzling than these results in Germanic languages. For example, using a cross-modal priming paradigm in Italian, Orsolini and Marslen-Wilson (1997) did not report any difference between effects observed for regular (e.g., *amarono*—*amare*, “they loved”—“to love”) and irregular sub-class (e.g., *presero*—*prendere*, “they took”—“to take”) verbs (but see Say and Clahsen, 2002). In contrast, findings in Portuguese have supported dual-route models, differentiating the lexicon and computational systems (Sicuro Corrêa et al., 2004; Veríssimo and Clahsen, 2009). These language-specific differences may reflect cross-linguistic specificities that are broadly noted in the morphological components (Beard, 1995; Chomsky, 1995; Marslen-Wilson, 2007).

Very few studies have assessed French inflectional categories to understand their lexical representation, access, and processing. Meunier and Marslen-Wilson (2004) used cross-modal and masked priming paradigms and showed that French inflected verbal forms present a facilitatory priming effect independently of their degree of stem regularity and allomorphy. In the cross-modal priming experiment, the priming effects were on the order of 51 ms for all types of verbs. In the masked priming experiment, significant priming effects varied from 16 ms up to 32 ms, depending on specific conditions. The authors concluded that morphologically related primes in French significantly facilitated response times (RTs) for all type of verbs, suggesting that decomposition takes place regardless of stem regularity. However, the variability of the effects observed in the masked priming experiment may suggest a more complex picture because the stem included in a prime such as *buvais* “I/you drank” overlaps minimally with the target *boire* “to drink.” Thus, if *[buv]ais* is decomposed, the remaining stem *[buv-]* does not overlap with the target stem *[boi-]*, as in the case of fully regular verbs (e.g., *[pass]ais* - *[pass]er* “I/you passed”—“to pass”). Therefore, the priming effects for idiosyncratic verbs, much like the system for their stem representation in the mental lexicon, remains open to question.

The use of priming techniques may cause specific experimental effects due to form-related processing that overlaps between priming and target (Allen and Badecker, 2002). One effective method to test verbal form decomposition is to measure the influence of the surface and cumulative frequencies on RT modulation (Taft and Forster, 1975; Taft, 1979, 2004; Burani et al., 1984; Colé et al., 1989, 1997; Schreuder and Baayen, 1995, 1997; Baayen et al., 1997; Meunier and Segui, 1999a; Domínguez et al., 2000). Therefore, we conducted a visual lexical decision task experiment in which we manipulated the cumulative and surface frequencies of verbs that differed in stem regularity.

In a seminal work in English word recognition, Stanners et al. (1979) showed that words matched in surface frequency have RTs modulated in the function of the cumulative frequency, with more frequent stems being recognized faster than less frequent ones. In Dutch, Schreuder and Baayen (1997) found the same type of results between high and low cumulative frequency words matched in the singular form in medium surface frequency. In a frequency study investigating Italian inflected verbs, Burani et al. (1984) obtained a significant difference between words with high and low cumulative frequencies matched in low surface frequency. Therefore, verbal inflection processing may be strongly related to cumulative frequency given its influence in the morphemic representation (Aronoff, 1994).

In French, as in other Romance languages, the right side of a verb has verbal suffixes that are paradigmatic realizations of morphosyntactic features of tense and agreement. The left side of the verb has a stem containing the root, which provides lexical information (Halle and Marantz, 1993; Kilani-Schoch and Dressler, 2005; Aronoff, 2012). In our experimental paradigm, we tested four verb types. (a) Fully regular verbs from the first group that have just one stem representation in the mental lexicon, which can be merged with the complete inflectional paradigm (Bybee, 1995). Thus, our hypothesis is that verbs are decomposed prior to lexical access, yielding a cumulative frequency effect between the forms of two regular verbs matched on their surface frequencies but with different cumulative frequencies. (b) Phonological change e/E verbs with orthographic markers are verbs from the first group but with two different predictable phonetic outcomes from the last <e> of the stem according to which suffix the stem is merged with (e.g., *[mèn]es* “you lead,” *[men]ons* “we lead”). They have an orthographic marker associated with the open phonetic production (i.e., <è>, <_ll> or <_tt>). (c) Phonological change o/O verbs without orthographic markers are verbs from the first group that present a predictable phonetic alternation in the last <o> of the stem but without any orthographic marker (e.g., *[dévOr]es* “you devour,” *[dévor]ons* “we devour”) (Kilani-Schoch and Dressler, 2005; Andreassen and Eychenne, 2013). For these two verbs types, the question is whether French speakers have two different phonetic representations of the stem in their mental lexicon or one phonological abstract underspecified representation of the stem that receives its phonetic form only in the spell-out of the word (Halle and Marantz, 1993; Marslen-Wilson and Zhou, 1999; Embick, 2013). This point was tested by contrasting the cumulative frequencies of different phonetic stem alternations. Finally, (d) idiosyncratic verbs from the third group have two or more unpredictable stem allomorphs to which the suffixes are merged (e.g., *[peu]t* “he/she can,” *[pouv]ons* “we can,” *[pu]* “could,” *[puiss]e* “I/he/she can”). Although previous results from Meunier and Marslen-Wilson (2004) suggested that these verbal forms are processed as fully regular ones, contrasting the cumulative frequencies of the different stems will allow us to test whether these idiosyncratic verbs have two or more different stem representations in the mental lexicon.

## Materials and methods

### Participants

Thirty-two adult native speakers of French between the ages of 18 and 32 (mean age: 20.31, 16 females) took part in this experiment as volunteers. All of the participants were right-handed, had normal hearing, normal or corrected-to-normal vision, no history of any cognitive disorder, and were undergraduate students at the *Université Lumière Lyon 2*. The participants did not know the purpose of the research and provided written consent to take part in the experiment as volunteers.

### Materials and design

We asked the participants to perform a lexical decision task on visually presented items. The participants gave their responses on a computer keyboard using two hands, a right-hand button “yes” to indicate existing words and a left-hand button “no” to indicate pseudowords. All of the words were chosen from the French corpus *Lexique 3* <http://www.lexique.org/> (New et al., 2004), which gives the frequency of the whole-word form (surface) and the frequency of the lemma per million words. In our study, the stem cumulative frequency was defined by summing the surface frequency of all inflected forms from each stem of interest.

To observe the different effects on the RTs as a function of the whole-word form and stem frequencies, we thoroughly manipulated and matched the cumulative and surface frequencies in the high and low ranges (Taft, 1979; Burani et al., 1984; Colé et al., 1989; Meunier and Segui, 1999a) as shown in Table 2.

**Table 2:** **Examples of experimental items according to verb type and frequency conditions**.

**Verb type**	**High cumulative frequency >140**		**Low cumulative frequency >80**	
	**High surface frequency >5**	**Low surface frequency >0.5**	**High surface frequency >5**	**Low surface frequency >0.5**
a. Fully regular	parlait	parliez	chante	chantez
b. Phono. e/E	répétait	répétions	répète	répètes
c. Phono. o/O	adorais	adoriez	adore	adores
c. Idiosyncratic	buvais	buviez	boivent	boives

Eighty stem pairs from the four verb types researched were selected, with 20 pairs for each verb type. All of the experimental words were inflected French verbs. We avoided inflected forms from the *passé simple*, the *subjonctif imparfait* and the participles because of their morphological productivity and specificity. The four verb types investigated were as follows: a. fully regular verbs, b. phonological change e/E verbs with orthographic markers, c. phonological change o/O verbs without orthographic markers, and d. idiosyncratic verbs. For the fully regular verbs, we did not use a stem pair from the same verb because these verbs have only one stem; instead, we used two different verbs with the same surface frequency. For the phonological change verbs, we calculated the stem cumulative frequency by summing all forms of each stem's phonetic realization. For the idiosyncratic verbs, we summed all forms of each allomorphic stem. We manipulated the cumulative and surface frequencies to match the four different conditions: two conditions with high cumulative frequency and high or low surface frequencies and two conditions with low cumulative frequency and high or low surface frequencies.

The experimental words in all verb types and conditions were not homographic with any other existent forms in French and had between six and eleven letters, between three and nine phonemes, and between one and four syllables. The words had an orthographic neighborhood size between one and three, as measured by the orthographic Levenshtein distance (OLD20), which compares words between all pairs of words in the lexicon, even with different lengths (Yarkoni et al., 2008). All of the experimental words were matched in their number of letters, number of phonemes, number of syllables, and OLD20 (see Table 3). The high cumulative frequency condition contained words with stem cumulative frequencies greater than 140, whereas the low cumulative frequency condition contained words with stem cumulative frequencies lower than 80. The high surface frequency condition had words greater than five form frequencies, whereas the low surface frequency condition had words fewer than 0.5 form frequencies. The complete list of experimental stimuli is available in the Supplementary Material.

**Table 3:** **Stimulus frequencies, letters, phonemes, syllables and OLD20**.

**Verb type**	**Cum**.	**Surf**.	**Cumulative frequency**	**Surface frequency**	**Letters**	**Phonemes**	**Syllables**	**OLD20**
a. Fully regular	High	High	278.38	6.34	7.90	5.10	2.30	1.90
	High	Low	278.38	0.34	8.10	6.15	2.85	2.03
	Low	High	62.04	6.23	7.95	5.50	2.50	1.95
	Low	Low	62.04	0.28	8.20	6.20	3.00	1.95
		
b. Phono. e/E	High	High	236.41	6.12	8.10	6.20	3.00	2.00
	High	Low	236.41	0.43	7.90	5.60	2.70	2.07
	Low	High	64.25	6.02	7.95	5.85	2.80	2.10
	Low	Low	64.25	0.35	8.20	6.30	3.00	2.01
		
c. Phono. o/O	High	High	215.87	5.96	8.20	5.90	2.85	1.98
	High	Low	215.87	0.19	8.15	5.65	2.70	2.11
	Low	High	60.29	6.12	7.90	5.50	2.00	1.95
	Low	Low	60.29	0.23	8.15	5.70	2.75	1.95
		
d. Idiosyncratic	High	High	258.84	6.09	8.35	6.80	2.65	2.08
	High	Low	258.84	0.36	8.20	6.45	2.50	2.15
	Low	High	61.85	6.18	8.20	6.40	2.65	2.00
	Low	Low	61.85	0.28	8.10	6.20	3.00	1.98

A set of 320 pseudowords was added to the 320 experimental items to produce the non-existent word response such that the experiment had 640 stimuli in total. The pseudowords were constructed by merging a non-existent but possible stem to an existent verbal inflectional suffix in French (pseudoverbs) (e.g., ^*^[[pors]ent], ^*^[[[lomb]i]ons]). Four different lists were constructed in a strict pseudo-random order to counterbalance the sequence of stimulus presentation between conditions. Each list was performed by eight participants. The lists had the following criteria: a. a stimulus was never preceded by another stimulus starting with the same letter, b. there were at maximum three words or pseudowords presented in sequence, c. there were at least 20 stimuli between words from the same lemma, and d. there were at least five stimuli between words/pseudowords with the same suffixes.

### Procedure

Participants were tested individually in a quiet room in the library at the *Université Lumière Lyon 2*. We used the E-Prime v2.0 Professional® (Schneider et al., 2012) software to construct the experiment as well as for stimulus presentation and data collection. Each trial followed the same sequence. First, a fixation point was displayed in the center of the screen for 500 ms at the same time as a “bip” sound was played. Immediately following the fixation offset, the target stimulus was displayed in the center of the 15” LCD screen in 18 point Courier New font in white letters against a black background. The target stimuli were presented in upper-case letters to avoid extra processing on the French accents. The RT recording started with the onset of the target stimulus presentation, which remained on the screen for 2000 ms or until the participant's response. After the target stimulus disappeared, the next trial started with the presentation of the fixation point. Participants were asked to perform a visual lexical decision task in which they decided whether the stimulus was an existent or a non-existent word (pseudoword) in each trial, pushing one of two keys as quickly and accurately as possible to indicate their choice. If the stimulus presented was an existent word, the participants were asked to push the right button; if the stimulus was a non-existent word (pseudoword), they were asked to push the left button. The experiment started with an instructional screen followed by a practice phase with eight stimuli. One break was provided in the middle of the experiment after 320 trials. The entire experiment lasted approximately 18 min.

## Results

For the experimental words, the by-participant average RT of correct acceptance was 695 (197) ms. Incorrect responses (9.62%) were removed from further analysis. Responses faster than 400 ms or slower than 1800 ms were also discarded (0.36%). Overall, 9.94% of the responses from the original data were discarded prior to statistical analysis.

RTs were logarithmically transformed to normalize their distribution. We conducted a mixed-effect model analysis (Baayen et al., 2008) on the data, with the logarithm of the RTs as the dependent variable in one analysis, and the accuracy as the dependent variable and a binomial distribution specified in another. Participants and Items were the random variables, and the Cumulative Frequency (high vs. low), Surface Frequency (high vs. low), and Verb Type (a. fully regular, b. phonological change e/E verbs with orthographic markers, c. phonological change o/O verbs without orthographic markers, and d. idiosyncratic) were the fixed-effect variables. The general RT means with their standard deviations in parenthesis and the error rates for each type of verb and each condition based on the by-participant analysis are displayed in Table 4.

**Table 4:** **Overall RT means, standard deviations, and error rates for each type of verb and condition**.

**Verb type**	**High cumulative frequency**				**Low cumulative frequency**			
	**High surface frequency**		**Low surface frequency**		**High surface frequency**		**Low surface frequency**	
	**RT (ms)**	**Error (%)**	**RT (ms)**	**Error (%)**	**RT (ms)**	**Error (%)**	**RT (ms)**	**Error (%)**
a. Fully regular	662 (171)	1.52	688 (180)	2.57	679 (168)	2.01	707 (197)	2.65
b. Phono. e/E	673 (186)	2.03	698 (187)	2.69	671 (176)	2.03	701 (201)	2.07
c. Phono. o/O	678 (181)	1.76	702 (198)	3.44	681 (179)	2.66	704 (168)	2.03
d. Idiosyncratic	681 (188)	1.87	697 (195)	2.50	698 (187)	2.54	716 (192)	2.73

### RT results

Overall, we found a significant effect for surface frequency [*F*_(1, 293)_ = 22.494, *p* < 0.001] and cumulative frequency [*F*_(1, 293)_ = 12.861, *p* < 0.01], but we did not find a significant effect between the different verb types [*F*_(3, 293)_ = 0.462, *p* = 0.709]. Regarding the general interactions, the only one that reached significance was between word type and cumulative frequency [*F*_(3, 293)_ = 8.238, *p* < 0.05]. This significant interaction effect will be further discussed by means of the different representations between regular and idiosyncratic verbs compared with phonological change verbs. Our main goal was to determine how the RT differences behaved for each verb type in terms of the surface and cumulative frequencies.

Planned comparisons given by the mixed effect model showed that fully regular verbs demonstrated a main effect for surface frequency, with high-frequency words being recognized faster than low-frequency words. This effect of 26 ms for high cumulative frequency words and 27 ms for low cumulative frequency verbs was significant [*t*_(292)_ = 2.942, *p* < 0.01]. There was also a main effect for cumulative frequency, with high-frequency words having faster responses than low-frequency words. This effect of 17 ms for high surface frequency verbs and 19 ms for low surface frequency verbs was also significant [*t*_(289)_ = 2.442, *p* < 0.05]. There was no significant interaction between cumulative and surface frequencies [*t*_(294)_ = 0.181, *p* = 0.857], suggesting that the two effects are independent of each other.

For phonological change e/E verbs with orthographic markers, there was a significant effect for surface frequency [*t*_(293)_ = 2.802 *p* < 0.05] of 25 ms in high cumulative frequency and 30 ms in low cumulative frequency verbs. However, there was no cumulative frequency effect [*t*_(290)_ = 0.521, *p* = 0.603], with a negative difference of −2 ms in high surface frequency verbs and only 3 ms in low surface frequency verbs, indicating that different frequencies in the stems of the phonological change e/E verbs with orthographic markers do not elicit different RTs for word recognition. There was no significant effect on the interaction between cumulative and surface frequencies [*t*_(291)_ = 0.535, *p* = 0.593].

For phonological change o/O verbs without orthographic markers, there was a significant effect for surface frequency [*t*_(294)_ = 2.406, *p* < 0.01], confirming the surface effect. This effect was 24 ms in high cumulative frequency verbs and 23 ms in low cumulative frequency verbs. However, there was no cumulative frequency effect [*t*_(292)_ = 0.078, *p* = 0.938], with a difference of only 3 ms in high surface frequency and 2 ms in low surface frequency verbs. There was no significant effect for the interaction between cumulative and surface frequencies [*t*_(294)_ = 1.358, *p* = 0.175].

Finally, idiosyncratic verbs showed a main effect in the surface frequency of 16 ms in high cumulative frequency and 18 ms in low cumulative frequency verbs. This effect was significant [*t*_(292)_ = 3.397, *p* < 0.01], confirming the surface effect. Importantly, there was also a significant main effect in cumulative frequency of 17 ms in high surface frequency verbs and 19 ms in low surface frequency verbs [*t*_(292)_ = 2.312, *p* < 0.05]. There was no significant effect on the interaction between cumulative and surface frequencies [*t*_(294)_ = 0.149, *p* = 0.882], suggesting that the surface and cumulative frequency effects are independent.

### RT discussion

Overall, we systematically observed a surface frequency effect for the four types of verbs tested; however, the picture for the cumulative frequency is different. Although its effect is clearly observed in the fully regular and idiosyncratic verb types, it does not appear in either type of phonological change verbs (with or without orthographic markers). This result explains why we found a significant interaction between verb type and cumulative frequency in the general analysis: regular and idiosyncratic verbs have different cumulative frequency behaviors compared with phonological change verbs. Because we did not find any cumulative frequency effect in this last verb type, phonetic alternations in the stem production may not be considered to be differently represented in the mental lexicon (Marslen-Wilson and Zhou, 1999; Embick, 2013). Therefore, these phonetic alternations do not result from different phonological representations but are most likely due to phonological abstract representations that receive their phonetic form after suffix computation in a later stage (Embick and Halle, 2005). To test this interpretation, we reconsidered the cumulative frequency for the stems as being the total cumulative frequency (i.e., the lemma frequency provided by the corpus), meaning the sum of both phonological changes for each type of verb (e.g., for the verb *lever* “to lift,” the cumulative frequency of the stem *[lev-]* of 347 per million was added to the cumulative frequency of the stem *[lEv-]* of 91 per million, resulting in a total cumulative frequency of 438 per million for all of its verb forms). We then conducted a *post-hoc* analysis through a new mixed-effect model (Baayen et al., 2008) that used the frequency values of surface and cumulative frequencies as continuous predictors. The logarithm of the RTs was the dependent variable, Participants and Items were the random variables, and the TotalCumulativeFrequency (numeric), SurfaceFrequency (numeric), and Verb Type (b. phonological change e/E verbs with orthographic markers, and c. phonological change o/O verbs without orthographic markers) were the fixed-effect variables.

For phonological change e/E verbs with orthographic markers in this analysis, there was a main effect of surface frequency [*t*_(291)_ = 2.495, *p* < 0.01]. Most importantly, there was a main effect of total cumulative frequency [*t*_(292)_ = 2.929, *p* < 0.01], confirming that the cumulative frequency of the phonological change verbs should not be considered separately between the different phonetic stem realizations. There was no significant effect for the interaction between total cumulative and surface frequencies [*t*_(287)_ = 1.055, *p* = 0.292].

For phonological change o/O verbs without orthographic markers, similarly to the phonological change e/E verbs, there was a main effect for surface frequency [*t*_(295)_ = 2.104, *p* < 0.01], and most importantly, there was also a main effect of total cumulative frequency [*t*_(288)_ = 2.238, *p* < 0.05], definitively confirming the total cumulative frequency effect in phonological change verbs. There was no significant effect for the interaction between total cumulative and surface frequencies [*t*_(292)_ = 0.868, *p* = 0.386], suggesting that both effects are independent.

These results confirm that phonological stem changes have only one abstract phonological underspecified representation in the mental lexicon (Marslen-Wilson and Zhou, 1999) and that the different phonetic productions are reflexes of phonological rules driven by the merger operation between the stem and suffixes (Embick, 2013).

### Error rate results

Fully regular verbs had an error rate of 8.12%, phonological change e/E verbs had an error rate of 8.83%, phonological change o/O verbs had an error rate of 9.88%, and idiosyncratic verbs had an error rate of 9.65%. High and low surface frequencies had error rates of 8.24% and 11.01%, respectively, whereas high and low cumulative frequencies had error rates of 7.79% and 11.45%, respectively. Overall, we did not find any significant error rate difference between the verb types [*F*_(3, 303)_ = 0.216, *p* = 0.885]. However, we did find significant error rate differences between the surface frequencies [*F*_(1, 303)_ = 5.202, *p* < 0.05], suggesting that words with higher surface frequencies are not only recognized more quickly but are also more easily recognized in visual stimulation as well as in the cumulative frequency [*F*_(1, 303)_ = 9.149, *p* < 0.01], suggesting that more frequent stems are more easily recognized than less frequent ones. No interaction reached significance, suggesting that verb type, surface frequency and cumulative frequency are independent.

## General discussion

In this work we investigated the mental representations and decomposability of French verbs. French is a rich morphological language in terms of lexical morphemes with fully regular stems, phonological stem changes, and idiosyncratic allomorphy in the stem (Kilani-Schoch and Dressler, 2005; Aronoff, 2012). We conducted an experiment in which the cumulative and surface frequencies were manipulated using high and low frequency conditions. Participants were asked to perform a lexical decision task as quickly and accurately as possible on visual items. The RTs and error rates were then analyzed as a function of our hypothesis.

We observed surface frequency effects for all types of verbs tested. More importantly, we observed cumulative frequency effects for the fully regular verbs from the first group and for the idiosyncratic verbs from the third group. The phonological change verbs presented slightly different results, yielding no cumulative frequency effect when the frequencies of the two phonetic stem forms were computed separately. However, the phonological change verbs yielded a significant total cumulative frequency effect when the cumulative frequency count included all of the conjugated forms of the verb, regardless of the phonetic form alternations. These results shed light on how verbal inflected forms are processed and how stems are represented in the mental lexicon depending of their type of regularity.

### Regularity

Fully regular French verbs from the first group have a single stem on which the verbal inflectional paradigm is based. Due to the paradigmatic system of verbal suffixes, it is extremely easy to identify and decompose the lexical morpheme (stem) from the inflectional endings containing morphosyntactic features (suffixes) (Bybee, 1995). Confirming our hypothesis, the significant cumulative frequency effect indicates that it is a predictive factor in word recognition, and its manipulation results in RT modulations (Taft, 1979). In this context, accordingly to Taft (2004, p. 747), the surface frequency effect “is explained in terms of the ease with which the information associated with the stem can be combined with the information associated with the affix.”

### Phonological changes

Unlike fully regular verbs, phonological change verbs have predictable alternations in their phonetic forms according to the phonological properties of the suffix to which the stem is merged (Embick, 2013). Therefore, the lack of an effect in the cumulative frequency between the phonetic alternation forms and the significant effect of total cumulative frequency confirms our hypothesis that verbs with phonological changes have an abstract phonological underspecified representation that is contacted during processing. Verbs with phonological changes are decomposed, and the different phonetic forms activate a single phonological underspecified stem (Marslen-Wilson and Zhou, 1999). An alternative hypothesis is that both different phonetic stems have a rule-based relation and only one of them is stored in the lexicon.

### Idiosyncrasy

For idiosyncratic verbs, similarly to the other verb types, the surface frequency effect should be interpreted as the recombination between the stem and affixes (Taft, 1979, 2004). Interestingly, we found a significant main effect in the cumulative frequency that can be broadly interpreted as differential access to different mental representations of the idiosyncratic stem allomorphs (Forster et al., 1987). However, this finding also suggests that even idiosyncratic known verbs are decomposed during visual recognition. These results are incompatible with models postulating that known words or irregular words are accessed by the direct whole-word route, such as the AAM (Caramazza et al., 1988) and the W&R (Pinker, 1999). Our results are in accordance with the earlier priming study in French on inflected verb recognition (Meunier and Marslen-Wilson, 2004). In French, even idiosyncratic verbs from the third group are decomposed due to the paradigmatic verbal inflectional system of suffixes (Bybee, 1995; Kilani-Schoch and Dressler, 2005).

### Decomposability and regularity

According to Rastle and Davis (2008), the recognition of polymorphemic words in visual modality begins with a morphological decomposition based on an analysis of orthography. Thus, because the orthographic regularity and relationships across the stems and the suffixes are extremely consistent in French (Bybee, 1995), we suggest that morphological decomposition is triggered more by the decomposability of verbal forms than by their regularity *per se*. Therefore, we argue that all French inflected verbs are first decomposed to their stem and suffixes and then these morphemes are accessed according to their cumulative frequency, generating the cumulative frequency effect. This decomposition activates lexical and morphosyntactic information systems, which are later recombined and verified for word recognition, generating the surface frequency effect. This assumption strongly supports the full-decomposition models (Halle, 1973; Taft, 1979, 2004; Halle and Marantz, 1993; Embick and Halle, 2005; Marantz, 2013) or the dual-route models, with a special emphasis on the combinatorial route (Wunderlich, 1996; Baayen et al., 1997; Orsolini and Marslen-Wilson, 1997; Clahsen, 1999). In this case, the bound-stems are stored in the mental lexicon, and inflected verbs share morphemic representations (such as roots, stems and suffixes) with all of the words from the same morphological family that have their own lexical entry representation.

### Nature of the representation

Studies conducted on Spanish have shown that word stress is defined by word structure, meaning that the morphemic nodes and the phonological characteristics of the merged morphemes are crucial for word stress (Oltra-Massuet and Arregi, 2005). The same analysis was conducted in Catalan (Oltra-Massuet, 1999), and similar assumptions were made by Andreassen and Eychenne (2013) in French (however, their argument was not deeply developed). Nevertheless, we suggest that word stress in French is strongly driven by word structure. In the case of verbs, word stress is defined by the tense and agreement nodes. The French iambic prosodic system is different from other Romance languages, which have a trochaic prosodic system. In this sense, it is the stressed syllable that defines the phonetic production in French phonological change verbs (Kilani-Schoch and Dressler, 2005; Aronoff, 2012). This means that the different phonetic stem productions of phonological change verbs are exclusively driven by prosodic rules, not by different morphological representations (Halle and Idsardi, 1996; Marslen-Wilson and Zhou, 1999; Embick, 2013). Accordingly to this assumption, our results showed that two phonetic alternation forms did not present any difference but activated a shared stem representation that is partly underspecified. Another possibility is that all morphemes are purely abstract and have no phonological content. Just after the morphemes are merged in the inflected word, the phonetic form is guided by phonological readjustment rules and is defined in a late insertion (Halle and Marantz, 1993; Embick and Halle, 2005; Marantz, 2013).

For idiosyncratic verbs, Meunier and Marslen-Wilson (2004) showed that different allomorphic stems have the same priming effects as fully regular verbs (e.g., *[boi]rons* “we will drink” and *[buv]ons* “we drink”) when priming their infinitive form (*[boi]re*, “to drink”). Our results significantly extend this investigation and suggest that allomorphic stems have different representations in the mental lexicon. Thus, the priming effect observed may be due to links between the different representations, or accordingly to Crepaldi et al. (2010) to a shared underlined representation in the lemma level (Forster et al., 1987; Allen and Badecker, 2002). Our results show that idiosyncratic verbs are decomposed and recognized through the specific stem representations of a single verb in the mental lexicon (Aronoff, 2012). Idiosyncratic stem allomorphs are represented in the mental lexicon as different bound-morphemes but are linked at a common abstract morphological level (Aronoff, 1994; Wunderlich, 1996; Clahsen, 1999). Thus, the time spent to recover a specific stem allomorph is modulated as a function of its cumulative frequency.

## Conclusion

The overall cumulative frequency effect is strong evidence that all inflected verbs in French are decomposed in visual modality independent of their stem regularity and phonological realization. Consequently, the surface frequency effect is interpreted as the result of the recombination between the lexical information of the stem and the morphosyntactic features of the suffixes (Taft, 1979, 2004). Taken together, our results can be explained by either an obligatory decomposition model (Halle and Marantz, 1993; Taft, 2004; Marantz, 2013) or a revised dual-route model similar to the MM model (Wunderlich, 1996), which posits completely combinatorial and internally structured representations.

### Conflict of interest statement

The authors declare that the research was conducted in the absence of any commercial or financial relationships that could be construed as a potential conflict of interest.
