# Supplementary material for: Decomposability and mental representation of French verbs
Source: Front Hum Neurosci. 2015 Jan 20;9:4. doi: 10.3389/fnhum.2015.00004 (PMC4299446; doi:10.3389/fnhum.2015.00004)
Supplement: Supplementary file 1 [file Table5.PDF]

Decomposability and mental representation of French verbs

Gustavo Lopez Estivalet[1,2]*, **Fanny Meunier**[1,2]

[1]CNRS UMR5304, Laboratoire sur le Langage, le Cerveau et la Cognition, Lyon, France
[2]Université de Lyon, Université Claude Bernard Lyon 1, Lyon, France

***Correspondence:** Gustavo Lopez Estivalet, Laboratoire sur le Langage, le Cerveau et la Cognition, Institut de Sciences Cognitives, 67 Boulevard Pinel, 69675 – Bron CEDEX, France.
gustavo.estivalet@isc.cnrs.fr

Supplementary material

a. Fully-regular verbs

Word	C+	S+	Word	C+	S-	Word	C-	S+	Word	C-	S-
aimions	795.61	6.55	aimeront	795.61	0.34	figurez	57.23	6.15	figurera	57.23	0.34
avancent	195,00	7.50	avancera	195,00	0.74	brillent	81.22	7.50	brillais	81.22	0.14
cherchez	448.99	7.36	cherchiez	448.99	0.74	baignait	41.42	6.62	baignons	41.42	0.14
donnerai	896.01	6.15	donneriez	896.01	0.34	reculons	69.05	6.62	reculera	69.05	0.14
entrent	398.38	7.30	entriez	398.38	0.20	détache	65.47	7.23	détachez	65.47	0.41
essayez	296.69	6.96	essayes	296.69	0.34	inspire	45,00	6.82	inspires	45,00	0.07
fermaient	197.16	5.27	fermera	197.16	0.34	utilisait	43.51	5.41	utilisons	43.51	0.07
frappent	168.31	5.34	frappons	168.31	0,00	tremblent	34.13	5.68	trembliez	34.13	0,00
gardais	257.50	5.74	garderas	257.50	0.61	discute	58.65	5.54	discutes	58.65	0.14
laisserai	851.55	5.81	laisseras	851.55	0.74	pardonnez	44.59	5.74	pardonnes	44.59	0.54
marchais	325.61	7.91	marcheras	325.61	0.27	insistait	67.03	7.97	insistons	67.03	0.20
occupent	219.80	7.30	occupiez	219.80	0.07	habille	67.36	7.97	habillez	67.36	0.20
oserait	155.54	5.34	oserons	155.54	0,00	agitent	89.19	5.54	agitera	89.19	0.07
oublierai	286.96	6.55	oubliiez	286.96	0.07	accusait	39.93	6.55	accusons	39.93	0.14
pleurais	163.31	5.07	pleurera	163.31	0.61	désirais	61.89	4.66	désireras	61.89	0.07
refusais	152.77	4.59	refuseras	152.77	0.27	organise	47.90	4.19	organisez	47.90	0.07
regardes	997.91	5.34	regardiez	997.91	0.54	dépassent	78.78	5.74	dépassais	78.78	0.41
roulent	163.45	6.28	roulera	163.45	0.34	examine	50.68	6.28	examines	50.68	0.07
serraient	207.50	5.81	serrerons	207.50	0.07	attaquait	70.41	6.01	attaquez	70.41	0.41
touchent	190.27	6.69	touchons	190.27	0.81	admirais	68.18	6.35	admires	68.18	0.41

b. Phonological change e/E verbs with orthographic markers

Word	C+	S+	Word	C+	S-	Word	C-	S+	Word	C-	S-
achetaient	122.98	2.16	achetions	122.98	0.20	achèterai	25.42	2.3	achètera	25.42	0.74
achevait	66.06	12.64	achevais	66.06	0.54	achève	15.41	11.22	achèvera	15.41	0.27
amenez	73.79	1.28	amenais	73.79	0.34	amènerait	20.15	2.16	amèneras	20.15	0.07
appelez	295.00	11.42	appeliez	295,00	0.07	appelles	170.33	8.72	appellerez	170.33	0.20
crevait	61.63	4.19	crevions	61.63	0.07	crèvent	19.94	5.47	crèvera	19.94	0.14
élevaient	79.22	4.73	élevais	79.22	0.61	élèvent	24.68	5.27	élèvera	24.68	0.34
emmenez	77.97	2.77	emmeniez	77.97	0.14	emmènerai	27.52	2.64	emmènerez	27.52	0.41
enlevez	60.76	2.23	enlevais	60.76	0.14	enlèvent	18.05	1.55	enlèverai	18.05	0.27
feuilletait	17.84	2.91	feuilletez	17.84	0.07	feuillette	3.65	3.51	feuillettes	3.65	0,00
jetais	277.45	3.04	jetons	277.45	0.27	jetterait	59.41	2.50	jetterez	59.41	0.20
levons	347.84	1.49	leviez	347.84	0.07	lèvera	91.57	1.89	lèveras	91.57	0.07
menaient	102.18	6.96	menions	102.18	0.14	mènent	35.56	7.57	mèneras	35.56	0.14
pesaient	51.52	3.99	pesions	51.52	0.14	pèsent	19.40	4.32	pèsera	19.4	0.54
projetait	27.04	6.69	projetais	27.04	0.74	projette	5.75	4.73	projettes	5.75	0,00
ramenaient	83.83	3.72	rameniez	83.83	0,00	ramènerait	25.42	2.03	ramèneras	25.42	0.14
rappelez	128.06	8.04	rappelons	128.06	0.41	rappelles	75.22	8.38	rappellerez	75.22	0.14
rejetait	40.47	5.74	rejetez	40.47	0.07	rejette	6.09	5.34	rejettes	6.09	0,00
relevaient	101.01	3.51	relevais	101.01	0.20	relèvent	23.94	2.57	relèves	23.94	0.20
renouvelait	16.84	1.62	renouvelez	16.84	0,00	renouvelle	2.58	1.62	renouvelles	2.58	0,00
semait	22.45	1.49	semaient	22.45	0.54	sèment	2.99	1.89	sèmera	2.99	0,00

c. Phonological change o/O verbs without orthographic markers

Word	C+	S+	Word	C+	S-	Word	C-	S+	Word	C-	S-
accrochais	83.51	1.01	accrochiez	83.51	0,00	accrochent	3.26	2.64	accrochera	3.26	0.07
adorais	24.34	2.30	adoriez	24.34	0.07	adorent	20.29	2.91	adores	20.29	0.27
affolait	13.12	2.70	affoliez	13.12	0,00	affole	5.28	4.19	affolera	5.28	0.07
approchez	158.51	2.43	approchiez	158.51	0.07	approchent	37.64	4.93	approches	37.64	0.61
bloquait	19.26	2.03	bloquais	19.26	0.20	bloque	3.86	3.04	bloquent	3.86	0.54
collaient	90.08	3.78	collais	90.08	0.34	collent	17.11	2.97	collerez	17.11	0,00
dévorait	29.72	4.32	dévorons	29.72	0.14	dévore	7.92	4.86	dévores	7.92	0.07
envolait	22.49	2.16	envolons	22.49	0,00	envolent	7.39	2.30	envoles	7.39	0,00
étonnais	81.64	3.58	étonnons	81.64	0.20	étonnerait	34.96	7.84	étonnerai	34.96	0.07
évoquais	57.59	2.03	évoquez	57.59	0.07	évoquent	15.96	2.57	évoquerez	15.96	0,00
flottaient	47.24	6.62	flottions	47.24	0.14	flottent	16.70	5.34	flotteras	16.70	0,00
frottait	40.02	8.38	frottiez	40.02	0,00	frotte	10.14	8.58	frottes	10.14	0.07
ignorons	105.88	1.28	ignoriez	105.88	0.61	ignores	2.57	1.42	ignorera	2.57	0.27
interrogeait	58.03	8.24	interrogions	58.03	0.20	interroge	15,00	12.36	interroges	15,00	0.20
moquez	34.26	1.15	moquons	34.26	0.07	moquent	16.09	2.36	moquerez	16.09	0.07
nommait	50.76	6.82	nommais	50.76	0.27	nomment	11.57	1.22	nommes	11.57	0.07
rapprochait	42.52	7.57	rapprochais	42.52	0.27	rapproche	12.18	8.11	rapprochera	12.18	0.41
sonnent	68.67	3.85	sonnais	68.67	0.41	sonnerait	23.92	1.08	sonneras	23.92	0.07
téléphonez	50.63	1.69	téléphonais	50.63	0.34	téléphonerai	16.44	1.35	téléphonent	16.44	0.68
volaient	70.55	5.20	volons	106,00	0.20	volent	18.13	5.61	volerez	84,00	0.07

d. Idiosyncratic verbs

Word	C+	S+	Word	C+	S-	Word	C-	S+	Word	C-	S-
apercevait	85.43	25.68	apercevrai	85.43	0.20	aperçoit	42.49	21.82	aperçoives	42.49	0.14
apprendra	107.50	5.07	apprendrez	107.50	0.74	apprenais	31.97	5.47	apprenons	31.97	0.68
boirai	148.18	1.55	boiras	148.18	0.74	buvions	54.12	2.36	buviez	54.12	0.14
connaissiez	335.26	2.84	connaisses	335.26	0.74	connaîtrait	160.28	2.77	connaîtrez	160.28	0.74
craignait	49.66	20.14	craignes	49.66	0,00	crains	38.18	17.64	craindra	38.18	0.07
devenais	376.50	6.15	deveniez	376.50	0.14	deviendrait	89.26	8.58	deviendras	89.26	0.95
envoyaient	131.57	2.91	envoyions	131.57	0.07	enverrai	10.27	2.70	enverras	10.27	0.47
mourait	169.60	11.35	mouriez	169.60	0,00	meurent	42.51	10.47	meures	42.51	0.14
obtenait	62.71	2.36	obtenons	62.71	0.41	obtient	7.31	3.18	obtiendra	7.31	0.41
parvenais	95.42	5.07	parveniez	95.42	0.07	parviens	7.31	5.88	parviendra	7.31	1.22
prévenait	58.46	2.23	prévenais	58.46	0.07	prévient	10.21	2.03	préviendra	10.21	0.2
recevaient	94.33	4.93	recevions	94.33	0.81	reçoivent	27.36	4.19	reçoives	27.36	0,00
rejoignent	40.97	5.07	rejoignais	40.97	0.34	rejoins	29.59	3.65	rejointes	29.59	0.54
reprends	114.73	7.16	reprendras	114.73	0.07	reprenaient	54.40	5.88	reprenions	54.40	0.68
retenait	103.52	15.34	retenions	103.52	0.34	retient	21.70	13.11	retiendra	21.70	0.54
revoyais	87.31	4.73	reverront	87.31	0.74	reverrai	18.51	4.05	revoyons	18.51	0.41
souvenaient	94.33	2.16	souvenions	94.33	0.27	souvienne	6.69	3.38	souviennes	6.69	0.27
surprend	28.46	7.09	surprenons	28.46	0.14	surprenait	13.53	7.30	surprendra	13.53	0.61
tenions	525.82	4.80	teniez	525.82	0.95	tiendra	193.04	4.59	tiendrez	193.04	0.74
valaient	82.31	5.27	valais	82.31	0.47	vaille	5.54	5.34	vaillent	5.54	0.21
